# An educational video to promote multi-factorial approaches for fall and injury prevention in long-term care facilities

**DOI:** 10.1186/1472-6920-14-102

**Published:** 2014-05-20

**Authors:** Laura B Dilley, Samantha M Gray, Aleksandra Zecevic, Gina Gaspard, Bobbi Symes, Fabio Feldman, Vicky Scott, Ryan Woolrych, Andrew Sixsmith, Heather McKay, Steve Robinovitch, Joanie Sims-Gould

**Affiliations:** 1 Centre for Hip Health and Mobility, 7/F, 2635 Laurel St, Robert H.N. Ho Research Centre, Vancouver, BC V5Z 1M9, Canada; 2 Faculty of Education, Simon Fraser University, Burnaby, British Columba, Canada; 3 Injury Prevention and Mobility Laboratory, Department of Biomedical Physiology and Kinesiology and School of Engineering Science, Simon Fraser University, Burnaby, BC, Canada; 4 Faculty of Health Sciences, School of Health Studies Arthur and Sonia Labatt Health Sciences, Western University, London, ON, Canada; 5 Fraser Health Authority, Surrey, BC, Canada; 6 British Columbia Injury Research & Prevention Unit and Ministry of Health, Office for Injury Prevention, Victoria, BC, Canada; 7 Gerontology Research Centre, Simon Fraser University, Vancouver, Canada; 8 Department of Family Practice, Faculty of Medicine, University of British Columbia, Vancouver, British Columbia, Canada

## Abstract

**Background:**

Older adults living in long term care (LTC) settings are vulnerable to fall-related injuries. There is a need to develop and implement evidence-based approaches to address fall injury prevention in LTC. Knowledge translation (KT) interventions to support the uptake of evidence-based approaches to fall injury prevention in LTC need to be responsive to the learning needs of LTC staff and use mediums, such as videos, that are accessible and easy-to-use. This article describes the development of two unique educational videos to promote fall injury prevention in long-term care (LTC) settings. These videos are unique from other fall prevention videos in that they include video footage of real life falls captured in the LTC setting.

**Methods:**

Two educational videos were developed (2012–2013) to support the uptake of findings from a study exploring the causes of falls based on video footage captured in LTC facilities. The videos were developed by: (1) conducting learning needs assessment in LTC settings via six focus groups (2) liaising with LTC settings to identify learning priorities through unstructured conversations; and (3) aligning the content with principles of adult learning theory.

**Results:**

The videos included footage of falls, interviews with older adults and fall injury prevention experts. The videos present evidence-based fall injury prevention recommendations aligned to the needs of LTC staff and: (1) highlight recommendations deemed by LTC staff as most urgent (learner-centered learning); (2) highlight negative impacts of falls on older adults (encourage meaning-making); and, (3) prompt LTC staff to reflect on fall injury prevention practices (encourage critical reflection).

**Conclusions:**

Educational videos are an important tool available to researchers seeking to translate evidence-based recommendations into LTC settings. Additional research is needed to determine their impact on practice.

## Background

Older adults who live in long term care (LTC) facilities are highly vulnerable to falls and fall-related injuries [1,2]. Most older adults living in LTC (60%) will sustain at least one fall per annum [3]. Given that falls are the leading cause of injury-related deaths and hospitalizations among older adults [2,4], preventing falls and fall-related injuries among this population is an urgent health priority. Recent research has recommended that falls injury prevention in LTC settings involve multifactorial approaches [5], which require conducting comprehensive assessments of the person and environment, establishing what factors create risk, and implementing a set of interventions that address those factors [6]. For example, removing environmental hazards, implementing an exercise programme and overseeing changes to medication regimes would together constitute a multifactorial fall prevention strategy [6]. Continuing education for LTC staff is a critical piece of fall prevention strategies in LTC settings, as it ensures that staff has access to information regarding best practices [7-9].

The Technology for Injury Prevention in Seniors (TIPS) program is a multi-year research program funded by the Canadian Institutes of Health Research (CIHR) that seeks to identify best practices in fall prevention and translate these into practical and sustainable strategies for LTC settings. TIPS used innovative technologies to determine the causes and circumstances of falls among older adults, including:

1.) Networks of digital video cameras and wearable sensors to record falls and fall mechanisms and

2.) Hip protectors and compliant flooring to reduce the risk for fall-related injuries.

As part of the TIPS study, digital video cameras were installed in common areas (dining rooms, lounges, and hallways) of two LTC facilities in British Columbia, Canada. Cameras recorded resident activities and any adverse events, such as falls. This footage provided unique evidence of the causes and mechanisms of falls within older adults’ everyday context [1]. Incorrect weight shifting (i.e., shifting of the body's center-of-gravity outside the base of support provided by the feet) was identified as the most common cause of falls, and these most commonly occurred when individuals were walking, sitting down, or standing [1].

Given the significant potential for TIPS to inform multi-factorial fall prevention strategies, and requests from LTC staff to view the video captured falls in efforts to improve their practices around fall and injury prevention, the research team (SKIPS: “Support Knowledge and Injury Prevention in Seniors”) was formed. The goal of SKIPS (also funded by CIHR) was to promote the uptake of TIPS research findings among staff in LTC settings, and included TIPS research team members (FF, JSG, BS, HM & SNR), geriatrics researchers (AS, RW & AZ) and nurses and administrative staff with experience in LTC settings (GG & VS). Given the unique opportunities provided by the video footage of falls, the project team decided to develop two educational videos for use in degree and diploma programs for LTC staff (e.g., nurses, care aides, etc.) and as part of continuing education in LTC settings. The decision to make videos was based on the availability of the fall capture videos and requests from LTC to develop fall and injury prevention educational materials that were easy to understand, easy to access and could be delivered on a coffee break (around 15–20 minutes).

The two videos were developed in conjunction with the SKIPS team and LTC staff. The videos integrate fall capture video footage and interviews with older adults, LTC staff, and fall prevention researchers and provide a practical, easy-to-understand approach to implement fall prevention recommendations in LTC settings. These educational videos draw upon principles of adult learning theory [8,10,11]. The videos provide educators with an easy to use tool that can be used in academic settings, LTC settings and as part of continuing and online education. The videos (available at: http://www.youtube.com/sfuTIPS) and accompanying curriculum for classroom and continuing education activities (available at: http://www.sfu.ca/tips), are designed to improve fall injury prevention in LTC settings through increased understanding of the causes of falls and how falls might be prevented. They also provide a seldom seen look at what actual falls look like. Most falls go unwitnessed so these educational videos provide a rare opportunity to stimulate discussion about real fall instances. Based on these educational videos, the objectives of this paper are to: 1) outline the unique aspects of these videos and; 2) provide an overview of the video development process to assist nurse educators (and others) who may wish to conduct similar projects, in future.

## Methods

### Development process: from pre-production to dissemination

In 2012, the SKIPS team embarked on a one-year project to develop two educational videos*, “Everybody Falls Sometime: An Introduction to the Prevention of Falls and Injuries in Long-Term Care”* and “*Evidence from Real-Life Falls: How to Manage Risk and Prevent Injuries in Long-Term Care*”, to support the implementation of evidence-based fall and fall related injury prevention training in LTC settings. Educational videos were identified as the ideal strategy given that video was a medium through which the previously acquired fall capture videos [1] could be showcased. In addition, visual media is critical in promoting engagement, critical awareness and deep learning among learners by modelling experiences directly relevant to their lives [12,13]. It was also determined that educational videos would be helpful because they could be easily accessed by nurse educators and students at any time of day of any location [12], and supported opportunities for continuous reinforcement, distance learning and reflection and engagement beyond the workplace. We also had feedback from LTC staff that had seen some of the video captured falls through presentations by our research team and had requested the opportunity to show these videos to their colleagues (and have discussion about them).

### Interdisciplinary team

An experienced adult educator with a background in visual arts was hired as the Project Coordinator (LBD) to develop the videos in collaboration with the TIPS study team. In addition, the Project Coordinator regularly consulted with healthcare professionals working in LTC settings, including clinical nurse specialists and nurse educators, to enhance the relevance of the video and supporting curriculum for LTC staff. The Project Coordinator also had access to transcripts from six focus groups with the LTC staff on their practices and learning needs with respect to fall and injury prevention. This interdisciplinary approach was rooted in the acknowledgement that, due to the complexities of fall injury prevention in LTC settings and the need for diverse perspectives to develop comprehensive fall prevention strategies. This approach recognized that fall injury prevention is beyond the scope of any single discipline, and that engaging diverse viewpoints allows for the development of a more holistic approach to learning [14,15].

### Learning needs assessment

The Project Coordinator conducted a learning needs assessment for fall injury prevention in LTC settings by drawing on findings from the focus groups, conversations with LTC staff and residents, peer-reviewed articles, and evidenced-based best practice guidelines available online. The project team determined that LTC staff needed further education regarding: (i) how to identify extrinsic and intrinsic risk factors for falls; (ii) how to implement multifactorial risk reduction strategies for fall injury prevention; and, (iii) ethical dilemmas relating to quality of life and autonomy in the context of fall injury prevention. These learning needs informed the development of the video so as to enhance its relevance to LTC staff and consider potential resource limitations in LTC settings.

To respond to these learning needs, SKIPS was guided by a conceptual approach that was designed to encourage practice reflection. This approach suggests that learners construct knowledge through a process of meaning-making that involves the hearing, telling and recognizing of stories [11,16]. Thus, in the introductory video, *“Everybody Falls Sometime”* older adults and long-term care staff shared stories of their experiences with falls and fall injury prevention. Learners are prompted to think about their own experiences in relation to the video [17]. In this regard, the learner makes sense of what they see and hear in the video by relating it to what they already know. Once learners engage in this process (referred to as ‘meaning making’), they are able to translate this new knowledge into their own stories. Through this process, the content of the story becomes more immediate, relevant and personal for the learner, which creates a deeper connection with the material [16,18]. In this instance, because the first video captures care aides and older residents of LTC, this process of meaning making is likely to be more poignant for these groups. The second video, “*Evidence from Real-Life Falls*”, emphasizes evidence-based recommendations for fall and injury prevention, and seeks to highlight steps that the motivated learner can take to minimize falls in LTC settings.

### Development process-meetings

The project team met four times over the course of the project. During an initial phone meeting, the project team finalized a project timeline and discussed the video’s objectives. Next, the Project Coordinator developed a video treatment, which outlined the video shoot location (i.e., one of the partnering LTC facilities), potential participants, approach to the video, and other technical aspects of creating the video. These dimensions of the final product were informed by initial project meetings, site visits to the partnering LTC facility, and lessons learned during the learning needs assessment.

The second meeting (in-person) involved ‘pitching’ the video treatment to the project team. Project team members were provided a detailed script that outlined the structure and content of the video in advance of the meeting. The script was revised during the meeting. Project team members viewed TIPS fall capture videos that were linked to key research recommendations and discussed how these might fit within the new video. The project team determined that two nurse educators (GG & VS) and two biomechanical engineers (SNR & FF) would appear in the video, and each would speak to a fall capture video and provide accompanying evidence-based practice recommendations.

At the next meeting (in-person) the project team reviewed a rough cut of the video. A feedback form was used to make recommendations that would guide the editing process. Following this meeting, a rigorous editing session was conducted to complete the video. At the final meeting (by phone), the team discussed the video dissemination strategy. As a part of this dissemination strategy, the Project Coordinator worked collaboratively with nurse educators on the project team (VS & GG) and clinical nurse specialists and nurse educators in both of the partnering LTC settings to develop lesson plans to accompany the videos. These lesson plans elaborated upon the videos by linking the narrative content to specific evidence-based recommendations identified in the TIPS project. Lesson plans were designed for LTC and academic settings. At various stages of the project, the project team consulted with nurse educators to ensure content and tone was consistent with the needs of LTC staff and relevant for preparing students enrolled in academic programs to provide care and support to older adults in LTC settings.

## Results

### The educational videos

The final deliverables were one 7 minute video and one 12 minute video, designed to be watched independently or together. *“Everybody Falls Sometime”* weaves together the stories of older adults and LTC staff in relation to falls and fall injury prevention. It seeks to prompt reflection on fall injury prevention practices among learners by validating their experiences and highlighting the social, emotional and physical impacts of falls. *Evidence from Real-Life Falls,* is more didactic and presents fall capture videos and interviews with fall injury prevention experts who link falls video footage to evidence-based practice recommendations. *“Everybody Falls Sometime”* may be particularly valuable to nurse educators who choose to emphasize the affective domain of learning (i.e., how we learn through feelings and emotions). *Evidence from Real-Life Falls* is tailored to the cognitive domain of learning (i.e., how learn based on facts and concepts). These videos are described below.

### “Everybody falls sometime”: an introduction to the prevention of falls and injuries in long-term care

To develop this video we adopted widely accepted tenets of adult learning theory; for example, the need to involve adults in the planning and evaluation of their instruction [10]. Therefore, LTC staff were invited to participate in the production of the video through the telling of their ‘fall injury prevention’ stories. Specifically, the Project Coordinator and a research assistant interviewed three LTC staff (two care aides and one licensed practical nurse) who focused on their experiences with fall and injury prevention in LTC settings. The interviews were conducted over three days in one of the partnering long-term care facilities. Semi-structured interviews were also conducted with three older adults who lived in this facility and who had experienced a fall. These individuals were asked questions regarding: 1.) how aging has impacted their mobility; 2.) what factors they believed contributed to their video captured fall and any subsequent falls; and, 3.) How they have been impacted by falls.

Participant narratives were used to tell the stories of LTC staff and older adults as experienced by staff and residents. The Project Coordinator in liaison with the larger team selected narratives to represent key themes that emerged during interviews with participants (i.e., circumstances under which unexpected shifts in weight occurred and environmental factors contributing to falls). Learning in adulthood is based on lived experience [11]. Thus, the candid nature of the interviews set within a LTC setting served as a powerful tool that staff identified with. Knowles and colleagues [10] suggest that adults are most interested in learning about subjects that have immediate relevance to their work and personal lives. The video sought to build upon this interest by emphasizing the lived experiences of LTC staff and older adults. This makes this video unique from other fall and injury prevention videos in that it is both for LTC staff and older adults and created and narrated by LTC staff and older adults.

### Evidence from real-life falls: How to manage risk and prevent injuries

The second video took a more traditional approach to instruction. Content was comprised of fall prevention experts who discuss four fall-related scenarios. Each case study begins with an introduction to address key issues related to cause and prevention of falls. Next, the video showcases a vivid, fall captured video that represents a common combination of cause and activity leading to a fall (e.g., tripping during walking, incorrect transferring during sitting down or rising, hit/bump, loss of consciousness). The onscreen case study discussion focused on: (1) what caused this fall; (2) how could the fall have been prevented; and, (3) what could be done to prevent injury in the event of such falls.

Researcher interviews in combination with fall capture videos create a synergy of auditory and visual learning styles. Dual-channel processing is more effective than single channel learning and learning is improved when instruction includes both visual and verbal information [19]. The inclusion of the real life fall video captured segments makes this video unique from other fall and injury prevention. This video gives the viewer a look at what is seldom seen, an actual fall in progress.

### Implementation of dissemination strategy

The completed videos were disseminated by the Project Team using a multi-dimensional strategy. This strategy engaged local community partners and included web-based distribution of the videos. In the local context, copies of the video and supporting curriculum were distributed to nurse educators in LTC settings across two health regions. Videos were screened at a local Geriatrics Services conference attended by more than 300 health care professionals and were featured at a Nurse Educator meeting hosted by the local health authority, attended by 18 clinical nurse educators and 4 clinical nurse specialists.

The project team undertook several activities to expand the reach of the video beyond the local area. First, videos were posted to YouTube along with keywords to attract web traffic. The project team also disseminated information regarding the videos using nursing and geriatrics listservs, newsletters, and social media. Further dissemination plans involve laying videos at LTC staff meetings, presentations at conferences (e.g. National Fall Prevention Conference, Toronto, May 2014).

## Discussion

The videos offer nurse educators a novel compliment or alternative to a traditional lecture format. The portability of the video provides learners and educators the opportunity to view the videos in a variety of settings, (i.e. in the work setting, classroom, at home and on their mobile devices). Educational media that is accessible, convenient, and learner-centered may improve the teaching and learning of content [12]. The videos are designed to enhance the learners understanding of mechanisms linked to falls in older adults -- an essential element to improve prevention strategies. The use of best practices and evidence-based content, as promoted in the videos, is a major priority with LTC and a requirement for accreditation. Our training tools are designed to respond to the needs of LTC care staff and administrators, health authorities, and ministries of health. We believe that video is a worthwhile and effective tool when those who will be on the receiving end of the education are involved in the production and narration of the videos. We also believe that the videos are but one mechanism to disseminate the findings from the larger TIPS program of research. The videos complement peer reviewed publications, presentations and lay research summaries and ensure that TIPS findings are accessible to a range of audiences.

These visual, teaching and learning tools will be used by LTC staff and integrated into training programs for new staff to promote understanding of mechanisms that cause falls and to improve fall risk assessment and prevention strategies. Nurse Educators might also use these visual methods to highlight other related topics in the curriculum or to tailor learning to specific issues that arise in work settings. Fall capture videos provide learners with the rare opportunity to observe react to and critically examine a real-life fall without the urgency that accompanies bearing witness to one. Lessons learned can be used to inform and perhaps enhance practice, in future.

### Limitations

This project encountered several challenges, and had several limitations, that should be considered. First, the relatively short time-frame for this project (one-year) limited the number and depth of consultations with LTC staff during the learning needs assessment. While the previously conducted focus groups had generated insights into the learning needs of LTC staff, they were not specifically focused on educational videos. Second, older adults who participated in the videos experienced multiple health challenges. These limited their ability to participate in video shoot over sustained periods of time. Finally, these videos were designed with a specific use in mind -- to effect positive change in fall and injury prevention in LTC settings only, and may not be relevant to other settings.

## Conclusion

The videos’ content weaves together the experiences of LTC staff and older adults and expert commentary by researchers to engage the learner. This approach, where the viewer is able to reflect on their own experiences in relation to what is happening in the video can contribute to improved practice [20]. The videos are less about “showing” LTC what to do but rather a mechanism to get them to think about what they are doing and how they might do it better (or differently) to help prevent fall and injuries. This approach, combined with expert commentary punctuates this ‘experiential or reflective learning’ with evidence-based fall injury prevention best practices. In conclusion, we believe that these videos have the potential to be more impactful than other fall and injury prevention videos for three reasons: 1) We involved end-users in the development of the videos from the inception stages; 2) Our approach was to guide leaners to think about their practice in relation to what they are seeing (as opposed to simply telling them what to do); and 3) we included very powerful and unique images of video footage of real life falls. Our next step is to assess whether these videos are effective learning tools in influencing fall and injury prevention practices amongst LTC staff.

## Competing interests

The authors declare that they have no competing interests.

## Authors’ contributions

All authors contributed to the conceptualization of this study and development of the videos. LBD wrote the first draft of the manuscript. All authors contributed to the critical revision of the manuscript and approved the final version.

## Pre-publication history

The pre-publication history for this paper can be accessed here:

http://www.biomedcentral.com/1472-6920/14/102/prepub
